# Relationship between Family Function, Anxiety, and Quality of Life for Older Adults with Hypertension in Low-Income Communities

**DOI:** 10.1155/2021/5547190

**Published:** 2021-09-27

**Authors:** Meng Zhang, Wenyan Zhang, Yu Liu, Meiliyang Wu, Jin Zhou, Zhongmin Mao

**Affiliations:** ^1^Nursing Department, Tongji Hospital of Tongji Medical College, Huazhong University of Science and Technology, Wuhan, China; ^2^Hanshui Bridge Street Community Health Center, Qiaokou District, Wuhan, China; ^3^Gutian Street Community Health Center, Qiaokou District, Wuhan, China

## Abstract

**Background:**

Effective functional family was beneficial for older adults' health, which may affect the quality of life (QoL) in hypertension patients. This study aimed to clarify the association between family function, anxiety, and QoL for older adults with hypertension in low-income communities.

**Methods:**

A questionnaire survey was conducted on 363 older adults with hypertension in low-income communities in Wuhan from September 2019 to November 2019. The relationships among the variables were examined by Pearson's correlation analysis. Predictor effects were tested using hierarchical multiple regressions, controlling for demographic characteristics. The structural equation model (SEM) was used to test the mediation effects of anxiety on the pathway from family function to QoL.

**Results:**

Family function was negatively correlated with the self-rating anxiety scale (SAS) score and positively correlated with the mental component score (MCS), but had no influence on the physical component score (PCS). Both PCS and MCS were negatively correlated with SAS. Anxiety was the negative predictor of MCS and PCS. Family function was the positive predictor of MCS, but had no influence on PCS. The path model indicated that anxiety significantly mediated the link between family function and QoL (*R*^2^ = 32.8%), but only partially.

**Conclusion:**

A significant correlation between anxiety, family function, and QoL was found. Anxiety had a partial mediating effect on the relationship between family function and QoL. Further research should focus on increasing the level of family function and reducing the perceived anxiety of older adults with hypertension to improve their QoL level.

## 1. Introduction

Hypertension is a health problem closely linked to poverty, accounting for 45% of heart disease deaths and 51% of stroke deaths, as reported by the WHO [1]. Relevant research showed that in the past 40 years, the problem of hypertension has spread from high-income countries to low-income countries [2]. A prospective study that included multiple high-, middle-, and low-income countries worldwide to investigate the prevalence, awareness, and control rate of hypertension in rural and urban populations concluded that low-income countries, low-income populations, and low-education populations had high blood pressure awareness rate, low treatment rate, and control rate [3]. In China, about 226 million adults were diagnosed with raised blood pressure, attributing to 24.6% of deaths and 12.0% of disability-adjusted life years [4]. The low control rate could be associated with complex symptoms of high blood pressure and deteriorated quality of life (QoL) [1]. QoL refers to the overall well-being of individuals, which is focused on the quality of a patient's life, rather than the length of survival [5]. Generally speaking, elderly with hypertension presented worsen QoL than older adults with no hypertension [6]. Especially, those who were living in the low-income communities had unequal access to care and medicines and obtained less comprehensive quality of health systems, presenting worsening QoL [2].

Family function was closely associated with QoL of the elderly. For most Chinese older adults, families are the primary source of social support, which is a protective determinant of health [7]. Functional families play a vital role in patients' performance with daily routine planning, such as meal planning, blood pressure monitoring, and medication adherence [8]. A considerable amount of literature has reported that family function had a positive correlation with the QoL of elderly patients. A study in northern Taiwan illustrated that better family function was correlated with the reduction of disabling symptoms and better QoL [9]. Another study in Filipino also indicated that higher family APGAR scores were significantly associated with better QoL for elderly patients [10]. However, many elderly adults with hypertension in low-income communities had mild or severe family dysfunction, and poor family function may be related to unsatisfactory QoL, but is scarcely discussed [11].

Emotional status was a commonly discussed factor in preliminary studies. The negative emotional reaction might lead to poor lifestyle behaviors, such as lack of physical exercise and poor medication adherence, which would further deteriorate QoL [12]. Moreover, anxiety as an important emotional status was frequently discussed in QoL. A cross-sectional survey with 205 participants illustrated that anxiety had a significant indirect effect on QoL through social support [13]. Another study indicated that anxiety and depression partially mediated the association between family caregiver's burden and QoL [14]. However, only limited studies proved the correlation between anxiety and QoL in elderly hypertension patients in low-income settings. In addition, anxiety was rare so as to explore its effect on family function and QoL. Therefore, we needed to explore the role of anxiety for a deep understanding of the relationship among them.

To address the aforementioned concerns, this study aims to investigate the QoL level of these older adults, explore the predictor of QoL, and clarify the relationships between family function, anxiety, and QoL, thus providing a reference for improving the elderly's QoL.

## 2. Methods

### 2.1. Study Design and Sample

A cross-sectional survey was performed from September 2019 to November 2019 in three low-income communities in Wuhan. Low-income communities are generally characterized as communities where geographic conditions can make mobility difficult. People in these locations typically have limited formal education, lack sanitary infrastructure, and are mostly unemployed [15]. The low-income communities were chosen from three regions of Wuhan, namely, Hanshui bridge community, Tanhualin community, and Zhiyin community, which were located in the Qiaokou District, Hanyang District, and Wuchang District, respectively. The inclusion criteria were: (a) age should be 60 years and older [16], (b) ability to communicate in Mandarin, (c) willingness to participate in this study, (d) diagnosed to have hypertension, and (e) dwelling in low-income communities for more than three months. The exclusion criteria were: (a) difficulty in providing answers, (b) having mental diseases, and (c) unconsciousness.

**Figure 1:**
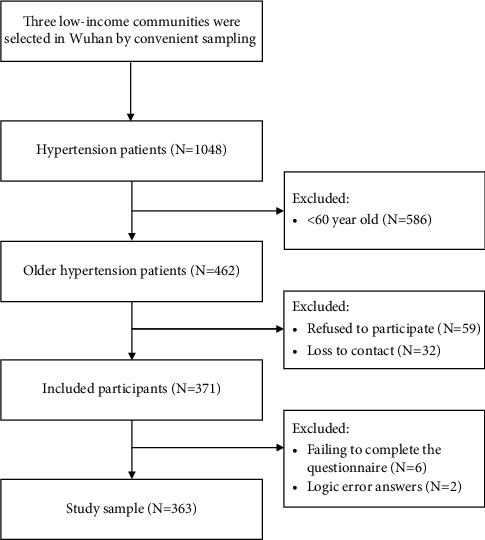
Sample selection flow diagram.

At the beginning of this study, we collected a name list of hypertension patients from the community councils. The council staff called the eligible elderly to ask if they would like to participate in the study. Then, the council staff introduced us to meet the elderly in the community council. The questionnaires were administered by two trained nurses in the community health service center to the old people or to the homes of the participants. The training included unified instructions and explanation of questionnaires in accordance with the oral habits of older adults in Wuhan. The authors informed the participants the research purpose, content, and significance before the formal research. An informed consent would be signed before the investigation. If the participants had visual impairment or unable to read or write, researchers would read every item and fill it out according to their oral description. Careful checks were done after gathering the questionnaire, and the questionnaires with missing items or invalid responses were returned to the participants to guarantee accuracy. A total convenience sample of 371 older hypertension patients were recruited in this study with a response rate of 80.30%. After 8 failures in completing the questionnaire and showing logic error answers, ultimately, the efficiency rate was 97.84% when 363 complete questionnaires were obtained (see Figure 1). The study was approved by the ethics committee of our hospital. An informed consent was obtained from all respondents. They were informed that their anonymity would be guaranteed, and the results obtained would be used only for this research. Complete confidentiality and security of the data related to the elderly were guaranteed.

### 2.2. Measurements

#### 2.2.1. Socioeconomic and Demographic Characteristics

A self-designed questionnaire was used to collect socioeconomic and demographic information, including gender, age, educational status, medical insurance, marital status, residential building, primary caregiver, average monthly medical expenses on hypertension, and average monthly household income.

#### 2.2.2. Family Function

The family function was assessed by the family APGAR Index, which was developed by Smilkstein in 1978 [17]. It was used to measure the satisfaction of family members in five domains: adaptability, partnership, growth, affection, and resolve. Each item was scored on a 3-point Likert scale from 0 (hardly ever) to 2 (almost always). The total score ranged from 0 to 10. Family function could be categorized as “good” (scores from 7 to 10), “moderate dysfunction” (scores from 4 to 6), or “severe dysfunction” (scores from 0 to 3) [17, 18]. The Chinese version of the family APGAR index was widely used with satisfactory validity and reliability [18, 19].

#### 2.2.3. Anxiety

The Zung self-rating anxiety scale (SAS), developed by Zung in 1971, was used to measure anxiety symptoms [20]. The SAS had 20 items, and each item was scored on a 4-point Likert scale. The standard score equaled 1.25 multiplied by the raw score. The higher score reflected a higher level of anxiety symptoms. A standard score exceeding 50 indicates that a person suffered from anxiety symptoms among the Chinese population [21]. The Cronbach's alpha of the scale was 0.81 [22].

#### 2.2.4. Quality of Life

The short-form health survey with 12 questions (SF-12) was used to assess QoL. It included 8 subscales (i.e., physical functioning, physical role, bodily pain, general health, vitality, social functioning, emotional role, and mental health) [23]. These 8 subscales were combined into physical component score (PCS) and a mental component core (MCS) [24]. The standard scores ranged from 0 to 100, and a score more than 50 indicated positive self-rated health [25]. Higher scores reflect better QoL [26].

#### 2.2.5. Statistical Analysis

The data were performed using SPSS 21.0. Data were tested for normality by using the Shapiro–Wilk test first. Continuous normally distributed data were presented as means and standard deviations, while categorical data were presented in frequencies and percentages. The scores of family APGAR, SAS, MCS, and PCS were continuous normally distributed variables as other studies [7, 27]. The reliability of family APGAR, SAS, MCS, and PCS was evaluated with Cronbach's alpha coefficients. All measures indicated satisfactory internal consistency above 0.75. The PCS and MCS among different categorical variables were evaluated by the *t*-test and one-way ANOVA. The Pearson correlation was conducted among family APGAR, SAS, MCS, and PCS.

Hierarchical multiple regression was performed with MCS and PCS as the dependent variables. The independent variables were entered in the regression model by block 1(sociodemographic characteristics), block 2(APGAR), and block 3(SAS) step by step. The method was used by entering sociodemographic characteristics in the first block to see if the main effects of these variables could influence work engagement, with family APGAR entered into block 2 to identify whether the APGAR score had a significant contribution once the main effects of sociodemographic characteristics have been considered; and SAS entered into block 3.

Mediation analyses were conducted with the M*plus* Version 7.4 software to calculate the mediation parameters for the total, direct, and indirect effects. Bootstrapping was used as it was more robust than the traditional Baron and Kenny approach or Sobel's test for testing mediation without the requirement of distributional assumptions. Additionally, bootstrapping can be used for making inferences about indirect effects in an intervening variable model, regardless of the complexity and number of the paths between predictors and outcomes. Bias-corrected 95% CI was used to assess the significance of direct and indirect effects. All analyses were two-tailed.

## 3. Results

### 3.1. Participants' Demographics

Of the remaining 363 respondents, the majority were female (67.77%) and 60∼79 years old (74.10%). Above half of the respondents had primary school level (21.49%) and junior high school level education (35.26%), and 73.83% had basic medical insurance for urban employees. The married participants accounted for 87.60%, and participants living in a building without elevator accounted for 74.65%. More than half (65.01%) of older adults had a primary caregiver, and 88.15% of older adults had retired. Additionally, about half of the participants expended 100∼500 RMB on hypertension per month (56.75%) and 2000∼3000 RMB for average monthly household income (52.07%) (see Table 1).

### 3.2. Correlations of Variables

The mean and standard deviation of APGAR, SAS, PCS, and MCS were as follows: 5.36 ± 2.92, 35.24 ± 7.58, 51.1 ± 24.66, and 70.87 ± 18.77. Pearson's correlation showed that APGAR was negatively correlated with SAS(*r* = −.211, *P* < 0.01) and positively correlated with MCS(*r* = .241, *P* < 0.01), but there was no significant relationship between APGAR and PCS (see Table 2). Both PCS(*r* = −.363, *P* < 0.01) and MCS(*r* = −.536, *P* < 0.01) were negatively correlated with SAS in significance.

### 3.3. Predictor of MCS and PCS

Table 3 provides significant results of the hierarchical multiple regression. The final model explained 36.2% of MCS and 31.4% of PCS. *R*^2^ change of each block for MCS was 19.2%, 4.3%, and 17%, while 29%, 0.3%, and 6.7% for PCS. As described in model 3 of Table 3, the negative predictor of MCS was marital status (widowed vs married), age, monthly medical expenses for hypertension, and anxiety (SAS). The negative predictor of PCS was gender (female vs male), medical insurance (others vs basic medical insurance for urban residents), marital status (widowed vs married), age, monthly medical expenses for hypertension, and anxiety (SAS). Family APGAR was the positive predictor for MCS, but had no influence on PCS (see Table 3).

### 3.4. Analyses of the Mediation Effect

**Figure 2:**
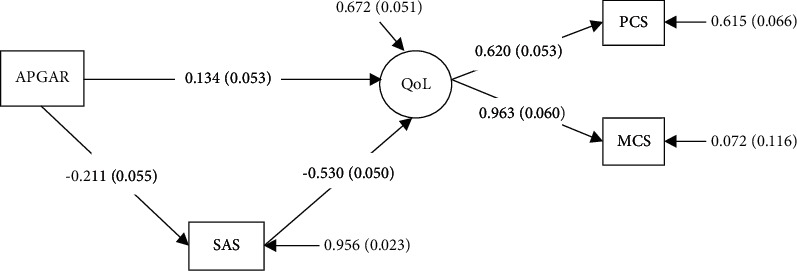
The mediation model. APGAR: family function; SAS: anxiety; QoL: quality of life; PCS: physical component score; MCS: mental component score. Chi-square = 2.893*∗*, *P*=0.089; RMSEA (root mean square error of approximation) = 0.072; CFI = 0.992; TLI = 0.953; SRMR (standardized root mean square residual) = 0.019.

To test the hypothesized model, the indirect effects of family function on QoL were examined with anxiety as potential mediating variables. All pathways within the model were significant as shown in Figure 2. Pathway 1: family function⟶QOL *β* = 0.134; pathway 2: family function⟶anxiety⟶QOL *β *=* *(−0.211)x(−0.530) = 0.112. In the final models, QOL was predicted indirectly by family function through anxiety (*β* = 0.112), indicating that better family function and lower anxiety contributed to a higher QoL. Furthermore, MCS (*β* = 0.963) contributed more than PCS (*β* = 0.620) in predicting QoL. Overall, the amount of variance explained by the model for QoL using the *R*^2^ was 0.328, showing that the model could explain 32.8% of the variance in QoL.

## 4. Discussion

This study investigated the current status and associated factors of QoL among Chinese older adults with hypertension in low-income communities, and also explored the mediating effect of anxiety on family function and QoL. The results showed that anxiety(SAS) was the negative predictor of MCS(*β* = −0.464, *P* < 0.01) and PCS(*β* = −0.290, *P* < 0.01). Family APGAR was the positive predictor of MCS(*β* = 0.140, *P* < 0.01), but no influence on PCS. Besides, the SME model illustrated that anxiety was a mediator on the pathway from the family function to QoL. These results improved our understanding of family function and anxiety in QoL, thus providing a reference to improving the QoL for older adults with hypertension in low-income communities.

In this study, the score of PCS(51.1 ± 24.66) and MCS(70.87 ± 18.77) presented moderate physical health and good mental health for older adults with hypertension in low-income communities. In addition, the physical health of older adults was worsened than that in middle or higher communities [28]. Our study found that these populations were the most vulnerable people in underserved areas. They lived with low physical health and needed more help from the health-care workers.

Both PCS and MCS were negatively correlated with SAS scores in significance in this study. Similar to a study conducted for patients with pulmonary hypertension, anxiety was significantly correlated with QoL [29]. Generally speaking, anxiety markedly impairs QoL in terms of both physical and mental health [29]. The presence of anxiety may further result in a worse quality of life, greater disability, and an increased rate of hospitalization [30].

Family function has been linked to many benefits of both physical and mental health [31]. Pearson correlation illustrated that family function was negatively correlated with SAS scores and positively correlated with MCS, but no influence on PCS, which indicated that family function was beneficial to mental health not physical health. Physical health was affected by multiple factors including age, education level, socioeconomic status, marital status, medical insurance, etc. [32]. Especially, the financial difficulty has considerable health implications for older adults' physical health, which cannot be changed with the help of low-income family members [33]. Age and gender were other irreversible factors influencing physical health [34]. Therefore, the family contributed more to patients' mental status, and indirectly improves patients' physical health, although the indirect factor was not significant in this study.

In agreement with previous studies [35], the regression model showed that hypertensive patients who were married, younger, with lower monthly medical expenses, and lower SAS scores had higher scores of MCS and PCS. The married patients had more support from their family members. However, the older patients faced many barriers, including increasing frailty, poor balance, chronic pain, polycomplication, disabilities, and other limitations [36]. This study also illustrated that higher monthly medical expenses for hypertension indicated lower MCS and PCS scores. Higher monthly medical expenses for hypertension indicated more serious condition of their illness. Along with the decline of physical function, social contact with friends and relatives withdraws accordingly [37]. Researchers should pay more attention to older patients with hypertension, especially those who cannot afford higher medical expenses.

SEM indicated that anxiety had a partial mediating effect on the relationship between the family function and QoL. In line with earlier research [27], the result indicated that better family function and lower anxiety contributed to a higher QoL, suggesting that family function and mental health were important for QoL of patients with hypertension. The functional family created an area of the individuals' sociocultural environment, serving as the main source of support to the elderly with hypertension, with the spouse and adult children having central roles [38]. Effective functional families provided informational, instrumental, and emotional support for older adults, which can inversely affect their mental status [38]. A study in South Korea similarly concluded that higher family function resulted in a lower degree of anxiety and depression [39].

In the SEM model, it was also observed that MCS (*β* = 0.963) may have more importance than PCS (*β* = 0.620) in predicting QoL for the older adults with hypertension in low-income communities. The result was correspondent with previous references [40]. The idea gave us a more comprehensive understanding of ways to facilitate older adults' QoL with hypertension. Mental health may contribute more than physical health to QoL. Predictors to promote the mental status of the elderly with hypertension should be highlighted, especially their family function. Further research should focus on increasing the level of family function and reducing the perceived anxiety of older adults with hypertension to increase their QoL level.

The present study had several limitations: a cross-sectional design was adopted by convenient sampling, which might not be generalized to other contexts. We enrolled older adults living in low-income communities for more than three months, which may be a selection bias. Moreover, the questionnaires were filled out by making the participants to recall their memories, so recall bias may occur. In addition, the recruited participants may be more interested in this study with a response rate of 80.30%.

In conclusion, this study investigated the relationship of anxiety, family function, and QoL among Chinese elderly with hypertension in low-income communities and examined the mediation model. We found a significant correlation between family function, anxiety, and QoL as described in the Results section. These findings are of great significance when providing the reference to improving the QoL for older adults with hypertension living in low-income communities. Interventions focused on increasing the level of family function, and reducing their perceived anxiety may increase their QoL level. Family empowerment researches were suggested to provide a new direction to improve the QoL of older adults with hypertension in low-income communities.

## Figures and Tables

**Table 1:** Older adults' characteristics.

		*N* (%)
Gender	Male	117 (32.32%)
	Female	246 (67.77%)

Age group	60–69	135 (37.19%)
	70–79	134 (36.91%)
	≥80	94 (25.90%)

Education	Illiteracy	63 (17.36%)
	Primary school	78 (21.49%)
	Junior high school	128 (35.26%)
	High school or technical secondary school	71 (19.56%)
	College	17 (4.68%)
	Bachelor degree or above	6 (1.65%)

Medical insurance	Basic medical insurance for urban residents	47 (12.95%)
	Basic medical insurance for urban employees	268 (73.83%)
	New rural cooperative medical scheme	33 (9.09%)
	Others	15 (4.13%)

Marital status	Married	318 (87.60%)
	Unmarried	9 (2.48%)
	Divorced	2 (0.55%)
	Widowed	34 (9.37%)

Residential building	Building with elevator	49 (13.5%)
	Building without elevator	271 (74.65%)
	Bungalow	43 (11.85%)

Having a primary caregiver	Yes	236 (65.01%)
	No	127 (34.99%)

Working status	On the job	18 (4.96%)
	Retirement	320 (88.15%)
	Unemployment	8 (2.21%)
	Others	17 (4.68%)

Average monthly medical expenses on hypertension (RMB)	<100	105 (28.93%)
	100∼500	206 (56.75%)
	>500	52 (14.32%)

Average monthly household income (RMB)	<2000	84 (23.14%)
	2000∼3000	189 (52.07%)
	>3000	90 (24.79%)

^∗^*P* < 0.05 (two-tailed). ^∗∗^*P* < 0.01 (two-tailed).

**Table 2:** Descriptive statistics and correlations of APGAR, SAS, PCS, and MCS.

	`*X* ± SD	APGAR	SAS	PCS	MCS
APGAR	5.36 ± 2.92	1			
SAS	35.24 ± 7.58	−0.211 ^*∗∗*^	1		
PCS	51.1 ± 24.66	0.085	−0.363 ^*∗∗*^	1	
MCS	70.87 ± 18.77	0.241 ^*∗∗*^	−0.536 ^*∗∗*^	0.597 ^*∗∗*^	1

^∗∗^Correlation is significant at the 0.01 level (2-tailed).

**Table 3:** Hierarchical multiple regression predicting MCS and PCS.

	Standardized beta coefficients					
	MCS			PCS		
	Model 1	Model 2	Model 3	Model 1	Model 2	Model 3
Block 1 demographic characteristics						
Gender (female vs. male)	−0.077	−0.105 ^*∗*^	−0.088	−0.132 ^*∗∗*^	−0.14 ^*∗∗*^	−0.129 ^*∗∗*^
Medical insurance (others vs. basic medical insurance for urban residents)	−0.035	−0.025	−0.020	−0.171 ^*∗∗*^	−0.168 ^*∗∗*^	−0.165 ^*∗∗*^
Marital status (widowed vs. married)	0.006	0.018	−0.103^*∗*^	0.076	0.079	−0.132 ^*∗∗*^
Age	−0.239 ^*∗∗*^	−0.225 ^*∗∗*^	−0.203 ^*∗∗*^	−0.339 ^*∗∗*^	−0.335 ^*∗∗*^	−0.321 ^*∗∗*^
Monthly medical expenses for hypertension	−0.231 ^*∗∗*^	−0.232 ^*∗∗*^	−0.151 ^*∗∗*^	−0.245 ^*∗∗*^	−0.245 ^*∗∗*^	−0.195 ^*∗∗*^
Block 2 APGAR		0.224 ^*∗∗*^	0.14 ^*∗∗*^		0.062	0.009
Block 3 SAS			−0.464 ^*∗∗*^			−0.29 ^*∗∗*^
Adjusted *R*^2^	0.140	0.183	0.362	0.244	0.245	0.314
Δ*R*^2^	0.192	0.043	0.170	0.29	0.003	0.067

^∗^*P* < 0.05 (two-tailed). ^∗∗^*P* < 0.01 (two-tailed).

## Data Availability

The raw data required to reproduce these findings cannot be shared at this time as the data also form part of an ongoing study.
